# Thymoquinone suppresses papillary thyroid carcinoma by impairing autophagic flux via the PDZK1IP1/HMGCS1 axis

**DOI:** 10.3389/fphar.2026.1966917

**Published:** 2026-10-26

**Authors:** Hanyang Lin, Ziqi Yan, Yuhan Shi, Zhaoyue Zhou, Qingxin Song, Xuanlu Zhang, Yuan Shao, Wei Liu, Bo Kou

**Affiliations:** 1 Department of Otolaryngology-Head and Neck Surgery, The First Affiliated Hospital of Xi’an Jiaotong University, Xi’an, Shaanxi, China; 2 Shaanxi Provincial People’s Hospital, Xi’an, Shaanxi, China; 3 Department of Human Anatomy, Histology and Embryology, School of Basic Medical Sciences, Xi’an Jiaotong University Health Science Center, Xi’an, Shaanxi, China; 4 Department of Thyroid, Breast and Vascular Surgery, The First Affiliated Hospital of Air Force Medical University (Xijing Hospital), Xi’an, Shaanxi, China; 5 Department of Vascular Surgery, The First Affiliated Hospital of Xi’an Jiaotong University, Xi’an, Shaanxi, China

**Keywords:** apoptosis, autophagic flux, HMGCS1, papillary thyroid carcinoma, PDZK1IP1, thymoquinone

## Abstract

**Background and Aim:**

Thymoquinone (TQ), the principal bioactive constituent of *Nigella sativa*, has antitumor activity in multiple malignancies, but its mechanism in papillary thyroid carcinoma (PTC) remains incompletely defined.

**Experimental Procedure:**

We evaluated the effects of TQ on PTC cell proliferation, apoptosis, autophagic flux, and tumor growth, and examined the PDZK1IP1/HMGCS1 axis.

**Key Results:**

TQ reduced viability and clonogenic growth in TPC-1 and BCPAP cells, increased apoptosis, downregulated PCNA and Bcl-2, and upregulated cleaved caspase-3, cleaved PARP, and BAX. TQ also increased autophagy-related vesicles and p62 and LC3-II levels. Tandem mRFP-GFP-LC3B reporter imaging showed accumulation of both yellow dual-positive and red-only LC3 puncta. Together with pharmacological modulation using bafilomycin A1, chloroquine, and rapamycin, these findings supported impaired late-stage autophagic processing. Integrated public PTC datasets and TQ-treated TPC-1 RNA-seq data identified *PDZK1IP1* as a candidate mediator. *PDZK1IP1* knockdown enhanced, whereas overexpression attenuated, TQ-induced proliferation inhibition, apoptosis, and autophagy-marker accumulation. HMGCS1 was identified as a downstream effector; its overexpression partially rescued these effects in both TPC-1 and BCPAP cells. In xenografts, TQ suppressed tumor growth, reduced Ki67, PDZK1IP1, and HMGCS1, and increased LC3 and p62.

**Conclusion and Implications:**

TQ suppresses PTC progression by impairing autophagic flux and downregulating PDZK1IP1/HMGCS1.

## 1 Introduction

Papillary thyroid carcinoma (PTC) accounts for the vast majority of thyroid cancer cases, and most patients achieve favorable long-term outcomes with standard-of-care management, including surgical resection, radioactive iodine therapy, and thyroid-stimulating hormone suppression (Brumfield et al., 2025; Wei et al., 2022). However, a subset develop locoregional recurrence, lymph node metastasis, or persistent disease, often with reduced sensitivity to conventional treatments. These clinical challenges underscore critical gaps in understanding the molecular pathways driving PTC progression and treatment resistance, highlighting the need for novel therapeutic targets and effective agents (Balentine, 2023; Chirra et al., 2026).

Thymoquinone (TQ), the main bioactive quinone constituent of *Nigella sativa*, exhibits well-documented antitumor activity in various human malignancies (Homayoonfal et al., 2021; Homayoonfal et al., 2022). Previous studies have linked TQ to impaired tumor cell proliferation, enhanced apoptotic cell death, and modulation of key oncogenic signaling pathways (Wilson et al., 2015; Pradeep Prabhu et al., 2024). While these findings identify TQ as a promising anticancer candidate, its specific biological effects in PTC and the downstream molecular mechanisms governing its activity in this disease, remain poorly understood (Ozturk et al., 2018).

Autophagy is an evolutionarily conserved lysosomal pathway that mediates the turnover of damaged organelles and misfolded intracellular macromolecules, maintaining cellular homeostasis under basal and stress conditions (Tang et al., 2020; Li et al., 2020). In cancer, autophagy exerts paradoxical, context-dependent effects: it can support tumor cell survival under metabolic or therapeutic stress, or drive growth suppression and immunogenic cell death, depending on tumor type and biological context (Niu et al., 2025; Wang et al., 2025). Static changes in autophagy-related protein markers do not reliably reflect dynamic autophagic activity; thus, assessing autophagic flux is essential to distinguish enhanced autophagosome biogenesis from impaired lysosomal degradation (Dong et al., 2023; Muralidharan et al., 2021). Accumulating evidence links dysregulated autophagic flux to altered proliferation, apoptotic sensitivity, and therapeutic response in malignant cells (Assi et al., 2026; Chen P. et al., 2024). However, whether TQ exerts its antitumor effects in PTC via disruption of autophagic flux remains unclear.

Among candidate molecular mediators, PDZK1IP1 (also known as MAP17) has been directly implicated in PTC progression. *PDZK1IP1* is upregulated in PTC and has been associated with adverse clinicopathological features and shorter progression-free survival; functional studies showed that its overexpression promotes PTC-cell proliferation and tumorigenicity, whereas silencing induces apoptosis and suppresses xenograft formation (Wang et al., 2022). A separate study reported that MAP17 promotes PTC growth and motility by interacting with NUMB and reducing p53 stability (Yu et al., 2021). In contrast, evidence for HMGCS1 in thyroid cancer remains limited. A transcriptomic comparison of PTC and normal thyroid tissue identified *HMGCS1* among PTC-associated differentially expressed genes annotated to cholesterol-response processes, but did not functionally validate its role (Zhu et al., 2013). Thus, whether PDZK1IP1 and HMGCS1 form a functionally connected pathway in PTC or mediate the response to TQ has not been established.

Metabolic reprogramming is a well-established hallmark of cancer, with tumor cells relying on coordinated metabolic and biosynthetic adaptations to sustain uncontrolled proliferation, survival, and stress resilience (Yang et al., 2023; Tufail et al., 2024). Transcriptomic profiling provides a powerful strategy to uncover molecular targets linking drug response to dysregulation of tumor-promoting pathways, including metabolic and autophagic signaling networks (Si et al., 2024). We hypothesized that TQ exerts its antitumor activity in PTC through coordinated regulation of autophagic flux and metabolism-related signaling axes, which had not been previously characterized in this context.

Hence, we systematically characterized the effects of TQ on PTC cell proliferation, apoptosis, and autophagic flux, and investigated the functional role of PDZK1IP1/HMGCS1 axis. Using *in vitro* functional assays, integrated transcriptomic analysis, molecular rescue experiments, and an *in vivo* PTC xenograft model, we tested the hypothesis that TQ suppresses PTC progression by impairing autophagic flux, with the PDZK1IP1/HMGCS1 axis serving as a key mediator of this effect.

## 2 Materials and methods

### 2.1 Reagents and antibodies

Thymoquinone (TQ; Cat. No. HY-D0803), chloroquine diphosphate (CQ; Cat. No. HY-17589), rapamycin (RAP; Cat. No. HY-10219), and bafilomycin A1 (BafA1; Cat. No. HY-100558) were acquired from MedChemExpress (Monmouth Junction, NJ, United States). TQ was dissolved in dimethyl sulfoxide (DMSO; Sigma-Aldrich, St. Louis, MO, United States; Cat. No. D2650) to prepare a stock solution and diluted to working concentrations immediately before use. Cell viability was assessed using the Cell Counting Kit-8 (CCK-8; Dojindo Molecular Technologies, Rockville, MD, United States; Cat. No. CK04). Cell proliferation was evaluated with the Click-iT Plus EdU Cell Proliferation Kit for Imaging (Alexa Fluor 488 dye; Thermo Fisher Scientific, Waltham, MA, United States; Cat. No. C10637), and apoptosis was analyzed using the BD Pharmingen FITC Annexin V Apoptosis Detection Kit I (BD Biosciences, San Jose, CA, United States; Cat. No. 556547). Lipofectamine RNAiMAX (Cat. No. 13778030) and Lipofectamine 3000 (Cat. No. L3000015; Thermo Fisher Scientific) were used for siRNA and plasmid transfection, respectively.

Primary antibodies against PDZK1IP1/MAP17 (rabbit monoclonal; Cat. No. ab156014; 1:5,000 for western blotting and 1:200 for IHC-P), GBP2 (rabbit polyclonal; Cat. No. ab247040; 0.2 μg/mL for western blotting), HMGCS1 (rabbit polyclonal; Cat. No. ab155787; 1:1,000 for western blotting and 1:200 for IHC-P), and Ki-67 (rabbit monoclonal, clone SP6; Cat. No. ab16667; 1:500 for western blotting and 1:200 for IHC-P) were obtained from Abcam (Cambridge, United Kingdom). Antibodies against PCNA (rabbit monoclonal; Cat. No. 13110), caspase-3 (rabbit polyclonal; Cat. No. 9662), cleaved caspase-3 (rabbit polyclonal; Cat. No. 9661), PARP (rabbit polyclonal; Cat. No. 9542), cleaved PARP (rabbit monoclonal; Cat. No. 5625), Bcl-2 (rabbit monoclonal; Cat. No. 4223), Bax (rabbit monoclonal; Cat. No. 5023), p62/SQSTM1 (rabbit polyclonal; Cat. No. 5114), LC3A/B (rabbit monoclonal; Cat. No. 12741), and GAPDH (rabbit monoclonal; Cat. No. 2118) were obtained from Cell Signaling Technology (Danvers, MA, United States) and used at 1:1,000 for western blotting. The LC3A/B antibody was used at 1:500 for IHC-P.

### 2.2 Cell culture

Human papillary thyroid carcinoma (PTC) cell lines TPC-1 and BCPAP were obtained from American Type Culture Collection (Manassas, VA, United States), and maintained in RPMI-1640 medium (Gibco, Thermo Fisher Scientific; Cat. No. 11875-093) supplemented with 10% fetal bovine serum (Gibco; Cat. No. 10099-141C) and 1% penicillin-streptomycin (Gibco; Cat. No. 15140-122). Cells were incubated at 37 °C in a humidified atmosphere with 5% CO_2_, and only logarithmically growing cells were used for experiments.

### 2.3 Cell viability assay

To evaluate the impact of TQ on PTC cell viability, we performed CCK-8 assays. Briefly, TPC-1 and BCPAP cells were seeded in 96-well plates at 4,000 cells per well, allowed to adhere overnight, then treated with a gradient of TQ concentrations for 24 or 48 h. CCK-8 reagent was added per the manufacturer’s instructions, and absorbance at 450 nm was measured using a BioTek Synergy H1 Hybrid Multi-Mode Reader (Agilent Technologies, Santa Clara, CA, United States). Viability was normalized to untreated controls, and half-maximal inhibitory concentration (IC_50_) values were calculated by nonlinear regression using GraphPad Prism.

### 2.4 Colony formation assay

Cells were seeded at low density (1,000 cells per well) in 6-well plates, allowed to attach, treated with TQ (0, 10, 20, 40 μM) or TQ combined with CQ or RAP. For gene manipulation, TPC-1 and BCPAP cells were transfected before drug exposure. Cultures were maintained for 14 days with medium refreshed every 3 days. Colonies were fixed with 4% paraformaldehyde (Solarbio, Beijing, China; Cat. No. P1110), stained with crystal violet (Sigma-Aldrich; Cat. No. C0775), and those with >50 cells were counted under a light microscope.

### 2.5 EdU incorporation assay

Cells were plated, subjected to the indicated treatments, then labeled with EdU per the manufacturer’s protocol. After fixation, permeabilization, and fluorescent labeling, cell nuclei were counterstained with DAPI. Fluorescence images were acquired using an EVOS M5000 Imaging System (Thermo Fisher Scientific, Waltham, MA, United States), and the proportion of EdU-positive cells was quantified from five randomly selected fields per group.

### 2.6 Apoptosis analysis by flow cytometry

Apoptotic cell death was quantified by flow cytometry using Annexin V-FITC/PI double staining. After treatment, TPC-1 and BCPAP cells were harvested, washed twice with ice-cold phosphate-buffered saline (PBS; Gibco; Cat. No. 10010–023), and resuspended in binding buffer. Staining was performed using the BD Pharmingen FITC Annexin V Apoptosis Detection Kit I per the manufacturer’s instructions. Data were acquired using a BD FACSCanto II Flow Cytometer with BD FACSDiva software version 8.0.3 (BD Biosciences, Franklin Lakes, NJ, United States) and analyzed using FlowJo version 10.8.1 (BD Life Sciences). Early and late apoptotic populations were combined to calculate the total apoptotic fraction for each sample.

### 2.7 Western blot analysis

Total protein was extracted from cells and xenograft tumor tissues using RIPA Lysis and Extraction Buffer (Thermo Scientific; Cat. No. 89900), supplemented with Halt Protease and Phosphatase Inhibitor Cocktail (100X; Thermo Scientific; Cat. No. 78440). Total protein concentration was quantified using the Pierce BCA Protein Assay Kit (Thermo Scientific; Cat. No. 23225). Equal amounts of proteins were separated by SDS-PAGE and electrotransferred to Immobilon-PSQ PVDF membranes (0.2 μm; MilliporeSigma, Burlington, MA, United States; Cat. No. ISEQ00010). Membranes were blocked with 5% BSA (Sigma-Aldrich; Cat. No. A7906) for 1 h and incubated with primary antibodies overnight at 4 °C. Membranes were then incubated with HRP-linked goat anti-rabbit IgG secondary antibody (Cell Signaling Technology; Cat. No. 7074; 1:2,000) for 1 h at room temperature. Protein bands were visualized using Amersham ECL Prime Western blotting Detection Reagent (Cytiva, Marlborough, MA, United States; Cat. No. RPN2232). Band densitometry was performed using ImageJ software (National Institutes of Health, Bethesda, MD, United States; https://imagej.nih.gov/ij/), and target protein levels were normalized to GAPDH.

### 2.8 Transmission electron microscopy

Ultrastructural changes in TPC-1 cells were examined by transmission electron microscopy (TEM). After the indicated treatments, cells were harvested and fixed with 2.5% glutaraldehyde prepared from 25% EM-grade glutaraldehyde solution (Electron Microscopy Sciences, Hatfield, PA, United States; Cat. No. 16220) at 4 °C for 2 h. After washing with 0.1 M phosphate buffer, samples were post-fixed with 1% osmium tetroxide prepared from a 4% aqueous stock solution (Electron Microscopy Sciences; Cat. No. 19140) for 1 h at room temperature. Samples were subsequently dehydrated through a graded ethanol series and embedded in SPI-Pon 812R epoxy resin (SPI Supplies, West Chester, PA, United States; Cat. No. 02660R-AB). Resin blocks were polymerized at 60 °C for 48 h. Ultrathin sections of approximately 60–80 nm were prepared and stained with 2% uranyl acetate solution (Electron Microscopy Sciences; Cat. No. 22400-2) for 15 min, followed by Reynolds’ lead citrate prepared from lead citrate trihydrate (Electron Microscopy Sciences; Cat. No. 17800) for 5 min. Sections were examined using a Hitachi HT7700 transmission electron microscope (Hitachi High-Technologies, Tokyo, Japan) operated at 80 kV. Autophagosome-like and other autophagy-related vesicular structures were identified based on their ultrastructural morphology.

### 2.9 Pharmacological modulation and tandem fluorescent LC3 reporter analysis

To investigate the role of autophagic flux in TQ’s antitumor activity, cells were treated with TQ alone, or combined with BafA1, CQ, or RAP. BafA1 (100 nM) was used for autophagic flux assessment, CQ (10 μM) and RAP (20 nM) were used alone or with TQ for functional assays. The effects were evaluated by TEM, western blotting, colony formation, and EdU incorporation assays.

To visualize LC3 trafficking, TPC-1 cells were infected with HBAD-mRFP-GFP-LC3 adenovirus (cat. no. HB-AP210 000; Hanbio, Shanghai, China) at a multiplicity of infection (MOI) of 50. At 24 h after infection, cells were treated with TQ (0 or 40 μM) for an additional 24 h. Following treatment, cells were fixed with 4% paraformaldehyde (cat. no. P1110; Solarbio, Beijing, China) at room temperature for 15 min, and nuclei were counterstained with DAPI (cat. no. 62248; Thermo Fisher Scientific, Waltham, MA, United States). GFP, mRFP, and merged fluorescence images were acquired using an LSM 880 confocal laser scanning microscope (Zeiss, Oberkochen, Germany) under identical acquisition settings. Five randomly selected fields were analyzed per group in each independent experiment, with approximately 18–22 cells per field and 90–110 cells analyzed per group. Yellow puncta (GFP+/mRFP+) and red-only puncta (GFP−/mRFP+) were quantified per cell using ImageJ software (National Institutes of Health, Bethesda, MD, United States). For each independent experiment, puncta counts were averaged across all analyzed cells, and the resulting experiment-level mean was used as one biological replicate for statistical analysis. Three independent experiments were performed.

### 2.10 GEO data collection and differential gene expression analysis

Publicly available gene expression datasets comparing PTC and paired normal thyroid tissues (GSE138198, GSE27155, GSE33630, and GSE9115) were retrieved from the Gene Expression Omnibus (GEO) database (https://www.ncbi.nlm.nih.gov/geo/). Analyses were performed in R version 4.5.2 (R Foundation for Statistical Computing, Vienna, Austria; https://www.r-project.org/). Expression matrices were downloaded with GEOquery version 2.78.0 (Bioconductor; https://bioconductor.org/packages/GEOquery), and probes were annotated to gene symbols using platform annotation files. After normalization, cross-dataset batch effects were corrected with ComBat in the sva package version 3.58.0 (Bioconductor; https://bioconductor.org/packages/sva). Differentially expressed genes (DEGs) between PTC and normal thyroid tissues were identified using limma version 3.66.0 (Bioconductor; https://bioconductor.org/packages/limma), with thresholds set at |log_2_ fold change| > 1 and adjusted P < 0.05.

### 2.11 RNA sequencing and integrated transcriptomic analysis

For RNA sequencing (RNA-seq), TPC-1 cells were assigned to three experimental groups: negative control (NC), low-dose TQ (TL), and high-dose TQ (QH). After TQ treatment, total RNA was extracted with TRIzol Reagent (Invitrogen, Thermo Fisher Scientific; Cat. No. 15596026). RNA integrity was verified on an Agilent 2100 Bioanalyzer with the Agilent RNA 6000 Nano Kit (Agilent Technologies, Santa Clara, CA, United States; Cat. No. 5067-1511). Sequencing libraries were prepared using the NEBNext Ultra II Directional RNA Library Prep Kit for Illumina (New England Biolabs, Ipswich, MA, United States; Cat. No. E7760) and sequenced on the Illumina NovaSeq 6000 platform (Illumina, San Diego, CA, United States). Raw reads were quality-filtered using fastp version 1.3.1 (https://github.com/OpenGene/fastp), and clean reads were aligned to the human reference genome GRCh38 (Ensembl release 108) using HISAT2 version 2.2.1 (http://daehwankimlab.github.io/hisat2/). Reads mapped to each gene were counted using HTSeq version 2.0.5 (https://htseq.readthedocs.io/), and gene expression was normalized as fragments per kilobase of transcript per million mapped fragments (FPKM). DEGs between NC and TQ groups were identified using DESeq2 version 1.50.2 (Bioconductor; https://bioconductor.org/packages/DESeq2), with thresholds set at |log2FoldChange| > 1 and P-value < 0.05.

To identify candidate genes mediating TQ’s antitumor activity in PTC, we integrated PTC-associated DEGs from GEO datasets with TQ-responsive DEGs from RNA-seq. Genes showing opposing expression patterns were shortlisted and overlaps were visualized with Venn diagrams. This identified *PDZK1IP1* and *GBP2* as top candidate genes; *PDZK1IP1* was prioritized for validation based on western blot confirmation.

### 2.12 GO and KEGG enrichment analysis

Gene Ontology (GO) and Kyoto Encyclopedia of Genes and Genomes (KEGG) enrichment analyses were performed on DEGs from the NC and high-dose TQ group comparison using clusterProfiler version 4.18.4 (Bioconductor; https://bioconductor.org/packages/clusterProfiler). Significantly enriched biological processes and pathways were extracted. Metabolism-related pathways were prominently enriched, leading us to select *HMGCS1* as a downstream target candidate for further investigation.

### 2.13 siRNA and plasmid transfection

For gene manipulation, TPC-1 and BCPAP cells were seeded in 6-well plates and grown to 60%–70% confluence before transfection. Small interfering RNA targeting *PDZK1IP1* (siPDZK1IP1; GenePharma, Shanghai, China; sense, 5′-GTT​GCA​ATC​GCC​TTT​GCA​GTT-3′; antisense, 5′-CTG​CAA​AGG​CGA​TTG​CAA​CTT-3′) and negative-control siRNA (GenePharma; sense, 5′-UUC​UCC​GAA​CGU​GUC​ACG​UTT-3′; antisense, 5′-ACG​UGA​CAC​GUU​CGG​AGA​ATT-3′) were transfected using Lipofectamine RNAiMAX (Thermo Fisher Scientific). *PDZK1IP1* overexpression plasmid (pcDNA3.1(+)-PDZK1IP1 containing the human *PDZK1IP1* coding sequence, NM_005764.4), *HMGCS1* overexpression plasmid (pcDNA3.1(+)-*HMGCS1* containing the human *HMGCS1* coding sequence, NM_002130.8), and the corresponding pcDNA3.1(+) empty vector were obtained from GenePharma and transfected using Lipofectamine 3000 (Thermo Fisher Scientific) according to the manufacturer’s protocol. At 48 h post-transfection, cells were harvested for western blot validation or used for functional assays.

### 2.14 Rescue experiments

To validate the functional interplay among TQ, PDZK1IP1, and HMGCS1, rescue experiments were performed in TPC-1 and BCPAP cells. After transfection with siPDZK1IP1, oePDZK1IP1, or oeHMGCS1, cells were treated with 40 μM TQ as indicated. Downstream assays included colony formation, EdU incorporation, and western blot analysis of proliferation, apoptosis, and autophagy markers. TEM was additionally performed in TPC-1 cells.

### 2.15 Xenograft tumor model

For the subcutaneous PTC xenograft model, twenty 4-week-old male BALB/c nude mice were acquired from the Animal Experimental Center of Xi’an Jiaotong University, Xi’an, China. All animal procedures were approved by the Biomedical Ethics Committee of Health Science Center of Xi’an Jiaotong University (approval No. XJTUAE2026-2059), and were performed in accordance with the Guide for the Care and Use of Laboratory Animals (NIH) and the Guidelines of the Biomedical Ethics Committee of Health Science Center of Xi’an Jiaotong University. TPC-1 cells were harvested, washed with PBS, and resuspended in serum-free medium. Each mouse received a subcutaneous injection of 5 × 10^6^ cells in a total volume of 200 μL into the right flank. Once tumors were palpable, 12 mice were included in the final analyzed cohort and randomly assigned to the control and TQ groups (n = 6 per group). The remaining eight animals were held in reserve and were not included in the analyzed cohort.

TQ was dissolved in 2% DMSO in PBS and administered at 20 mg/kg via intraperitoneal injection three times per week (approximately every other day), for a total of nine administrations; controls received an equal volume of vehicle on the same schedule. Tumor length and width were measured every 3 days with calipers, and volume was calculated as (length × width^2^)/2. Body weight was recorded at each measurement. On day 21, mice were euthanized by carbon dioxide inhalation using a gradual-fill displacement rate of 30%–70% of the chamber volume per minute, followed by cervical dislocation to confirm death. No anesthetic agents were used during subcutaneous tumor cell inoculation, routine intraperitoneal dosing, or tumor measurement. The euthanasia procedure was performed in accordance with institutional animal care guidelines and the American Veterinary Medical Association (AVMA) Guidelines for the Euthanasia of Animals. Xenograft tumors were excised, photographed, weighed, and collected for immunohistochemistry and western blot.

### 2.16 Immunohistochemistry

Tumor tissues were fixed in 10% neutral buffered formalin (Sigma-Aldrich; Cat. No. HT501128), embedded in paraffin, and sectioned at 4 μm. Sections were deparaffinized, rehydrated, and subjected to antigen retrieval in 10 mM sodium citrate buffer (pH 6.0). Endogenous peroxidase activity was quenched with 3% hydrogen peroxide, and non-specific binding was blocked with 5% BSA (Sigma-Aldrich; Cat. No. A7906). Sections were incubated overnight at 4 °C with primary antibodies against Ki-67 (Abcam; Cat. No. ab16667; 1:200), LC3A/B (Cell Signaling Technology; Cat. No. 12741; 1:500), PDZK1IP1 (Abcam; Cat. No. ab156014; 1:200), and HMGCS1 (Abcam; Cat. No. ab155787; 1:200). Sections were then incubated with ready-to-use SignalStain Boost IHC Detection Reagent (HRP, Rabbit; Cell Signaling Technology; Cat. No. 8114) for 30 min at room temperature. Immunoreactive signals were visualized using the ImmPACT DAB Peroxidase (HRP) Substrate Kit (Vector Laboratories, Newark, CA, United States; Cat. No. SK-4105), and nuclei were counterstained with Hematoxylin Solution, Gill No. 1 (Sigma-Aldrich; Cat. No. GHS132). Brightfield images were captured using an Olympus BX53 microscope (Olympus, Tokyo, Japan), and the proportion of positively stained cells was quantified from five randomly selected fields per section.

### 2.17 Statistical analysis

All quantitative data are presented as mean ± standard deviation (SD) from at least three independent experiments. Statistical analyses were conducted using GraphPad Prism 9.0 (GraphPad Software, San Diego, CA, United States). For the tandem mRFP-GFP-LC3 assay, the experiment-level means from three independent experiments were used for statistical analysis. Differences between two groups were assessed by unpaired Student’s t-test, and multiple-group comparisons were performed by one-way ANOVA followed by an appropriate *post hoc* test. Longitudinal tumor-volume and body-weight data were analyzed using two-way repeated-measures ANOVA, with treatment as the between-subject factor and time as the within-subject factor. The Greenhouse-Geisser correction was applied to the repeated time factor, followed by Sidak’s multiple-comparisons test comparing the control and TQ groups at each time point. P < 0.05 was considered statistically significant.

## 3 Results

### 3.1 TQ inhibits proliferation and induces apoptosis in PTC cells

We first characterized the antiproliferative activity of thymoquinone (TQ) in two human papillary thyroid carcinoma (PTC) cell lines, TPC-1 and BCPAP. The chemical structure of TQ is shown in Figure 1A. CCK-8 assays revealed that TQ reduced cell viability in a concentration- and time-dependent manner (Figure 1B). The half-maximal inhibitory concentration (IC_50_) values were 47.7 μM (24 h) and 16.3 μM (48 h) for TPC-1 cells, and 37.7 μM (24 h) and 23.3 μM (48 h) for BCPAP cells, respectively. This reduced viability corresponded with impaired clonogenic growth: colony formation assays exhibited a dose-dependent decrease in colony number after treatment with certain concentrations of TQ (Figure 1C). EdU incorporation assays confirmed a marked decline in the fraction of EdU-positive proliferating cells upon TQ exposure (Figure 1D).

**FIGURE 1:**
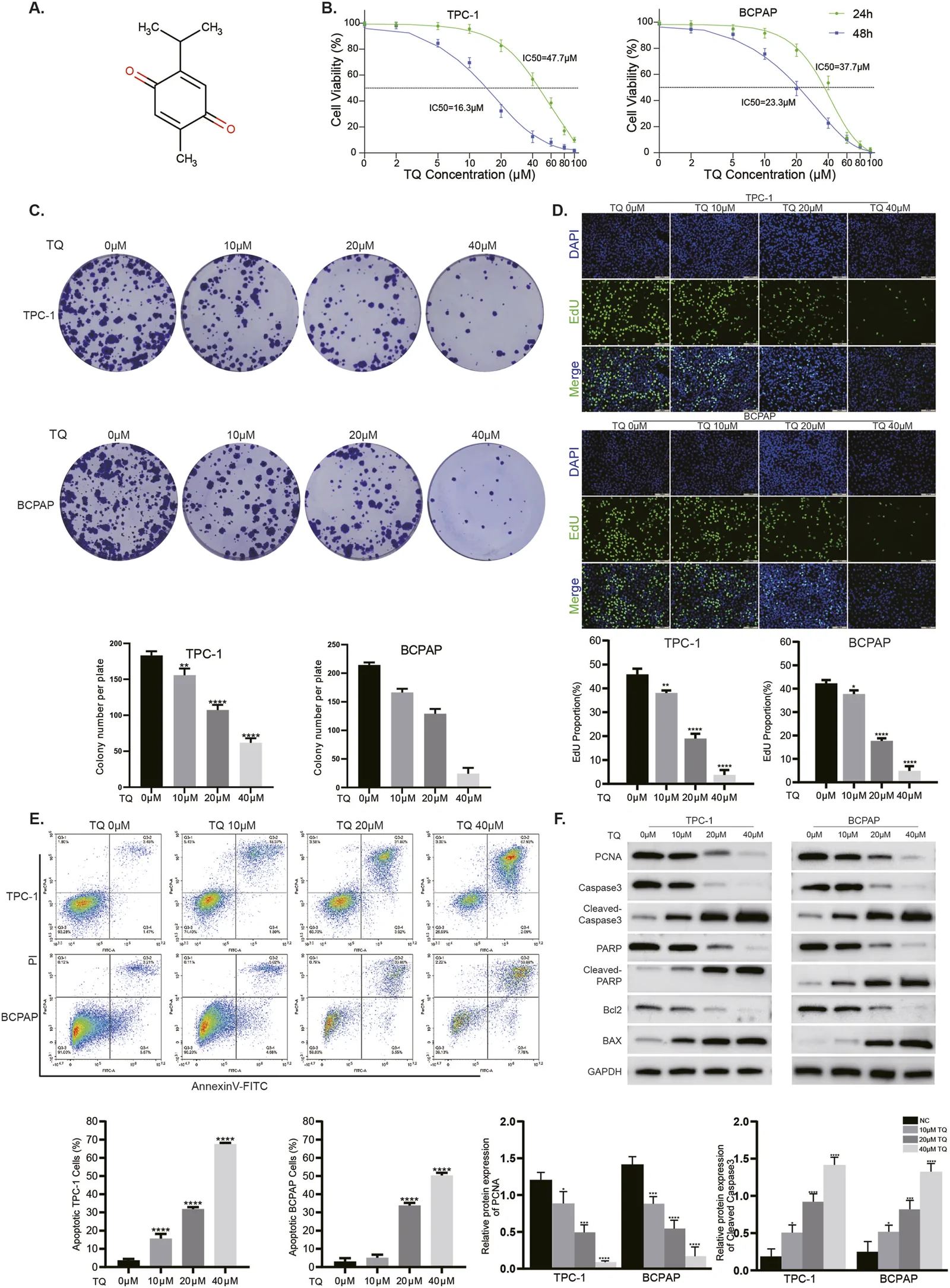
Thymoquinone suppresses proliferation and induces apoptosis in papillary thyroid cancer cells. **(A)** Chemical structure of thymoquinone (TQ). **(B)** CCK-8 assay of TPC-1 and BCPAP cells treated with TQ for 24 h and 48 h. **(C)** Colony formation assays. **(D)** EdU incorporation assays. **(E)** Flow cytometric analysis of apoptosis by Annexin V-FITC/PI staining. **(F)** Western blot analysis of proliferation- and apoptosis-related proteins in TPC-1 and BCPAP cells treated with TQ. Data are presented as representative images and quantification.

We next examined whether TQ-induced growth suppression was accompanied by apoptosis induction. Annexin V-FITC/PI flow cytometry revealed a dose-dependent increase in the total apoptotic fraction in both TPC-1 and BCPAP cells upon TQ treatment (Figure 1E). Western blot analysis further validated these changes: TQ downregulated the proliferation marker PCNA and anti-apoptotic protein Bcl-2, while upregulating cleaved caspase-3, cleaved PARP, and pro-apoptotic BAX (Figure 1F).

### 3.2 TQ triggers autophagic vesicle accumulation and impairs autophagic flux in PTC cells

To explore whether autophagy contributes to the cellular response to TQ, we assessed ultrastructural and biochemical changes in autophagy-related pathways following TQ treatment. Transmission electron microscopy (TEM) uncovered a remarkable accumulation of vacuolar and autophagosome-like structures in TQ-treated TPC-1 cells compared with untreated controls (Figure 2A). Tandem mRFP-GFP-LC3B reporter imaging further showed significant increases in both yellow dual-positive puncta (GFP+/mRFP+) and red-only puncta (GFP-/mRFP+) after TQ treatment (Figure 2B), indicating accumulation of LC3-positive autophagic compartments. Biochemically, TQ dose-dependently increased the protein levels of p62 and LC3-II in both TPC-1 and BCPAP cell lines (Figure 2C).

**FIGURE 2:**
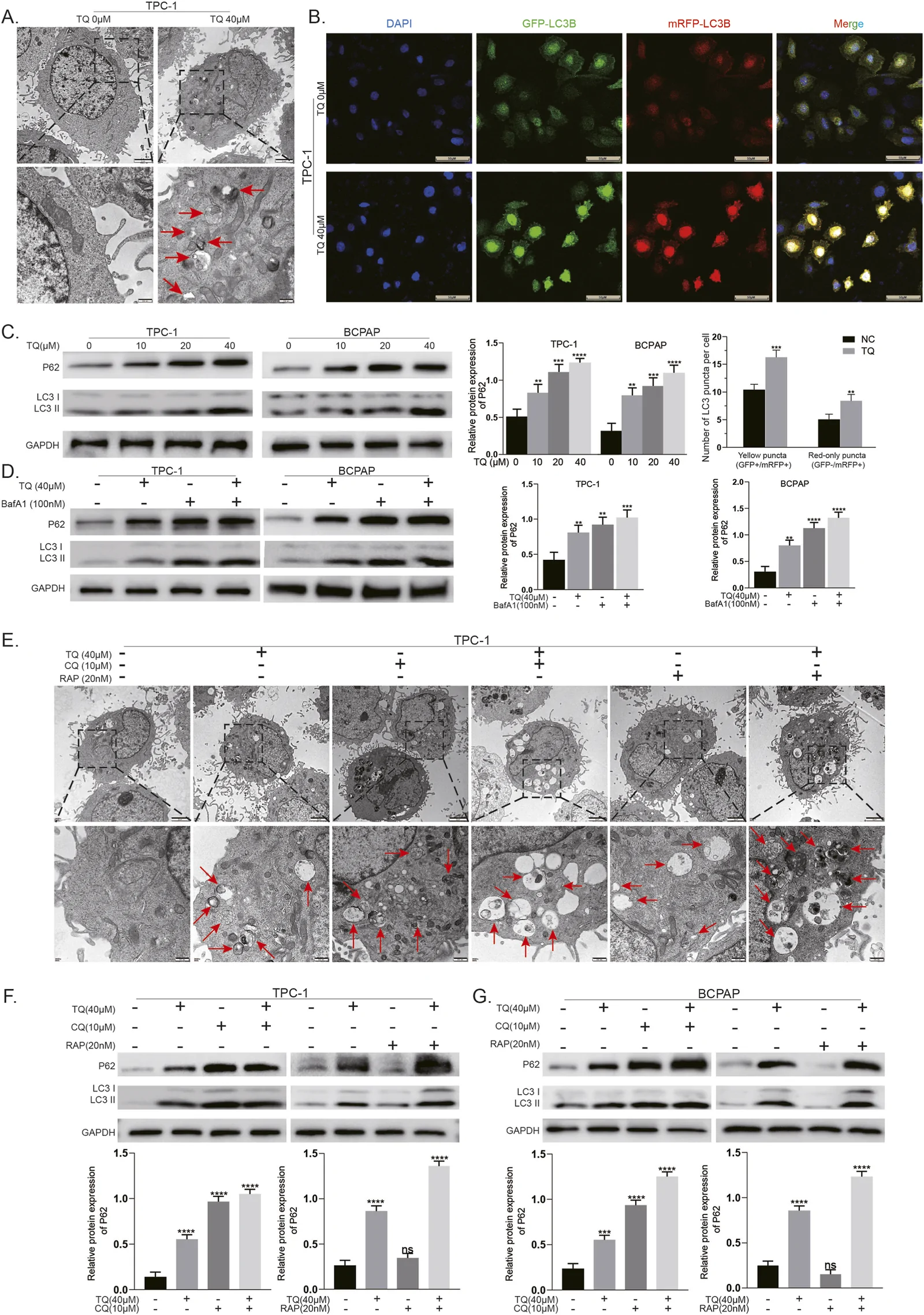
TQ induces autophagic vesicle accumulation and disrupts autophagic flux. **(A)** Transmission electron microscopy (TEM) of TPC-1 cells treated with TQ. **(B)** Tandem mRFP-GFP-LC3 reporter imaging and quantification of yellow puncta (GFP+/mRFP+) and red-only puncta (GFP−/mRFP+) in TPC-1 cells treated with 0 or 40 μM TQ. Five randomly selected fields and approximately 90–110 cells were analyzed per group in each experiment. Scale bars, 50 μm. Quantitative data are presented as mean ± SD from three independent experiments. **(C)** Western blot analysis of p62 and LC3 in TPC-1 and BCPAP cells treated with increasing concentrations of TQ. **(D)** Western blot analysis of p62 and LC3 in cells treated with TQ in the presence or absence of bafilomycin A1 (BafA1). **(E)** TEM of TPC-1 cells treated with TQ alone or combined with chloroquine (CQ) or rapamycin (RAP). **(F,G)** Western blot analysis of p62 and LC3 in TPC-1 **(F)** and BCPAP **(G)** cells under the indicated conditions.

Given that concurrent upregulation of p62 and LC3-II is consistent with impaired autophagic degradation, we next evaluated autophagic flux using three pharmacological modulators: the late-stage autophagy inhibitors bafilomycin A1 (BafA1) and chloroquine (CQ), and the autophagy activator rapamycin (RAP). In both cell lines, co-treatment with BafA1 and TQ further augmented p62 and LC3-II accumulation compared with TQ alone (Figure 2D). TEM imaging of TPC-1 cells revealed robust accumulation of autophagy-related vesicles following treatment with TQ or CQ alone, with a more pronounced phenotype in the TQ and CQ co-treatment group; by contrast, RAP treatment yielded a distinct ultrastructural pattern (Figure 2E). Western blot analysis confirmed these findings: CQ enhanced TQ-induced p62 upregulation in both TPC-1 and BCPAP cells, while RAP did not recapitulate this effect (Figures 2F,G). Collectively, the tandem reporter, p62/LC3-II accumulation, and pharmacological modulation support disturbed late-stage autophagic processing rather than simple autophagy activation, while indicating that autophagic maturation is not completely abolished.

### 3.3 Pharmacological modulation of autophagic flux regulates the antiproliferative and proapoptotic activity of TQ

To define the functional role of TQ-induced autophagic flux impairment, we treated PTC cells with TQ combined with CQ or RAP. In colony formation assays, TQ alone markedly suppressed clonogenic growth in both TPC-1 and BCPAP cells, and this effect was further enhanced by CQ co-treatment (Figures 3A,B). Conversely, RAP partially rescued clonogenic growth in TQ-treated cells.

**FIGURE 3:**
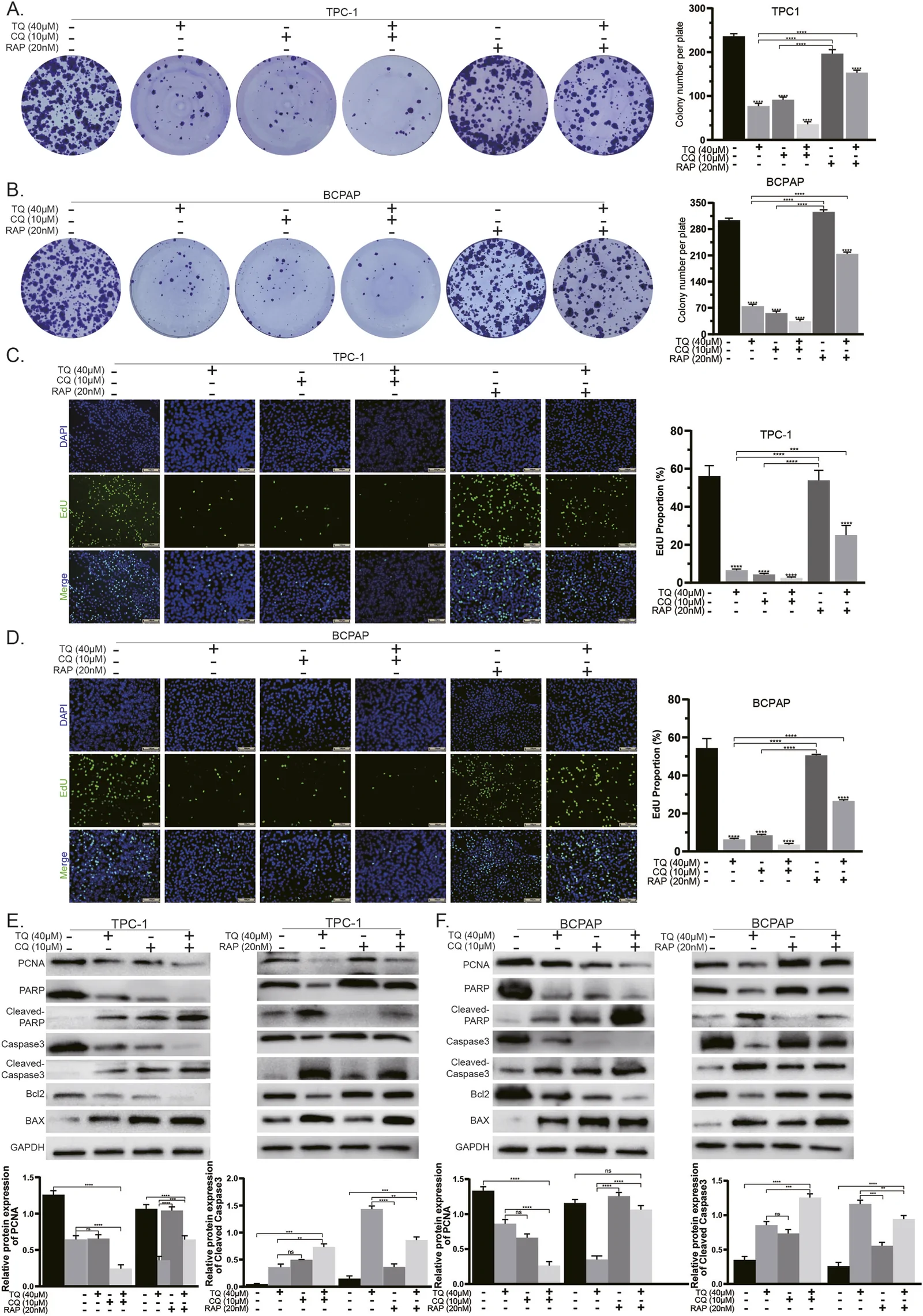
Pharmacological modulation of autophagy alters the antiproliferative and proapoptotic effects of TQ. **(A,B)** Colony formation assays of TPC-1 **(A)** and BCPAP **(B)** cells upon TQ treatment with or without CQ or RAP. **(C,D)** EdU incorporation assays of TPC-1 **(C)** and BCPAP **(D)** cells as indicated. **(E,F)** Western blot analysis of PCNA, cleaved caspase-3, PARP, Bcl-2, and BAX in TPC-1 **(E)** and BCPAP **(F)** cells.

These findings were recapitulated in EdU incorporation assays: TQ reduced the fraction of EdU-positive proliferating cells in both cell lines, an effect exacerbated by CQ and partially reversed by RAP (Figures 3C,D). Western blot analysis validated these phenotypic changes at the molecular level: relative to TQ alone, TQ co-treatment with CQ further downregulated PCNA and upregulated cleaved caspase-3 in both cell lines, while RAP partially abrogated these TQ-induced molecular changes (Figures 3E,F). These data indicate that TQ-induced impairment of autophagic flux directly contributes to its antiproliferative and proapoptotic effects in PTC cells.

### 3.4 Integrated transcriptomic profiling identifies *PDZK1IP1* as a key mediator of TQ’s antitumor activity in PTC

To uncover the molecular mechanisms underlying TQ’s antitumor activity in PTC, we integrated public PTC datasets with our RNA sequencing (RNA-seq) data from TQ-treated TPC-1 cells. We first analyzed four Gene Expression Omnibus (GEO) datasets comparing PTC and adjacent normal thyroid tissues. After batch effect correction, inter-dataset variation was minimized (Figure 4A), and differential expression analysis identified genes significantly dysregulated in PTC (Figure 4B).

**FIGURE 4:**
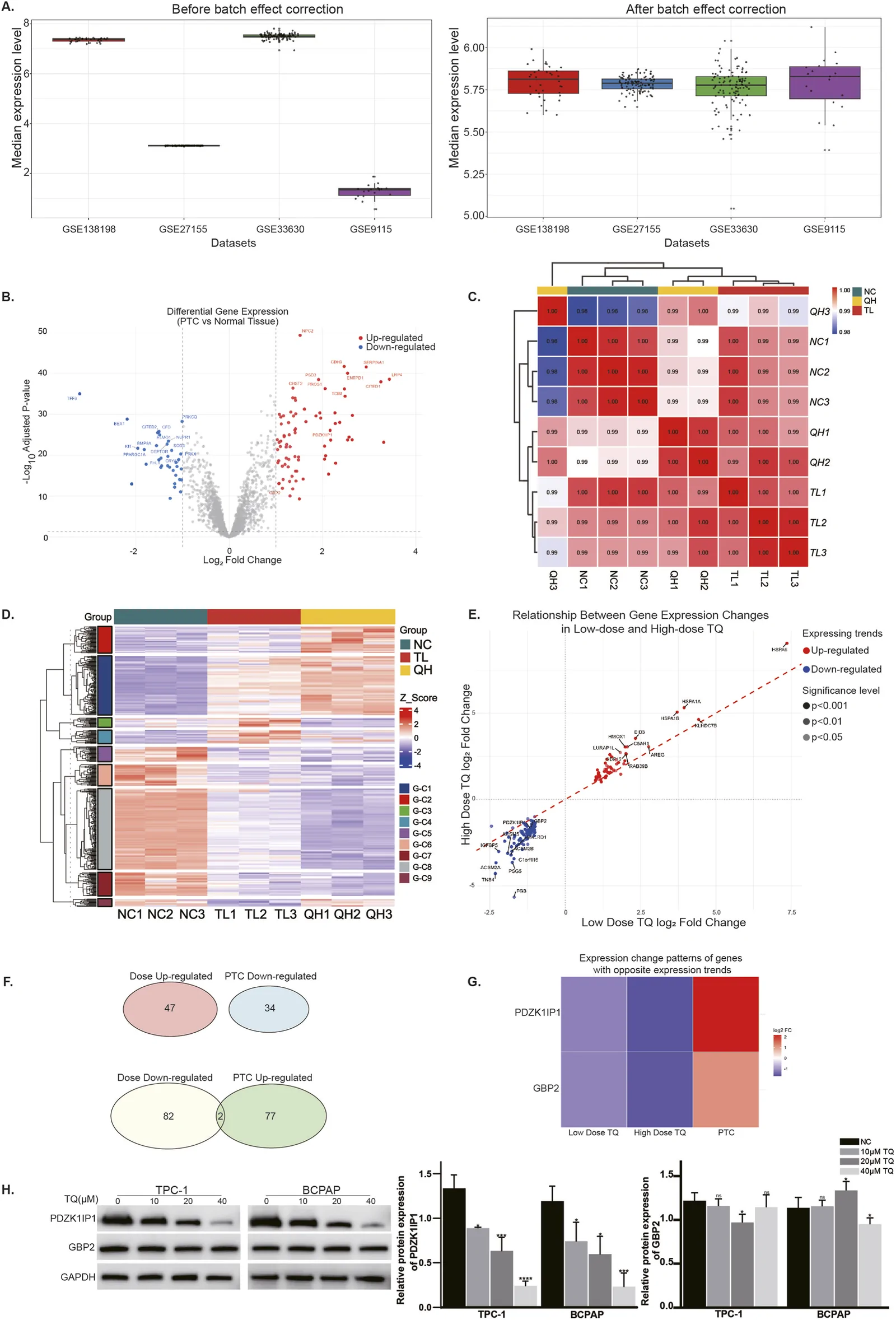
Integrated transcriptomic analysis identifies *PDZK1IP1* as a candidate mediator of the TQ response. **(A)** Box plots of GEO datasets before and after batch-effect correction. **(B)** Volcano plot of differentially expressed genes between papillary thyroid carcinoma (PTC) and normal thyroid tissues in the integrated GEO analysis. **(C)** Correlation heatmap of RNA-sequencing samples from the negative control (NC), low-dose TQ (TL), and high-dose TQ (QH) groups. **(D)** Heatmap of differentially expressed genes in TPC-1 cells upon TQ treatment. **(E)** Correlation analysis of expression changes between low- and high-dose TQ groups. **(F)** Venn diagram showing overlap between GEO-derived PTC-associated genes and TQ-responsive genes with opposite expression trends. **(G)** Identification of *PDZK1IP1* and *GBP2* as candidate genes. **(H)** Western blot validation of PDZK1IP1 and GBP2 in TPC-1 and BCPAP cells treated with TQ.

We next performed RNA-seq on TPC-1 cells treated with low-dose (TL) or high-dose (QH) TQ. Sample correlation analysis confirmed high reproducibility among biological replicates (Figure 4C), and unsupervised clustering clearly separated control from TQ-treated groups (Figure 4D). Direct comparison of TL and QH groups showed that most TQ-responsive genes were regulated similarly at both doses, with larger changes at the higher concentration (Figure 4E).

To narrow down candidate gene mediators, we intersected PTC-dysregulated genes from GEO datasets with TQ-responsive genes from our RNA-seq data, prioritizing those with opposing expression trends (Figure 4F). This intersection yielded two top candidates: *PDZK1IP1* and *GBP2*, both dose-dependently regulated by TQ, with more pronounced changes at the higher dose (Figure 4G). Western blot validation showed that PDZK1IP1 was more robustly and consistently downregulated by TQ in both TPC-1 and BCPAP cells than GBP2 (Figure 4H). We therefore selected PDZK1IP1 for subsequent functional validation.

### 3.5 PDZK1IP1 mediates TQ-induced proliferation suppression, apoptosis, and autophagic flux impairment

To functionally validate the role of PDZK1IP1 in TQ’s antitumor activity, we performed loss- and gain-of-function experiments in TPC-1 cells. Western blotting confirmed successful knockdown and overexpression of PDZK1IP1 (Figure 5A). Colony formation assays revealed that PDZK1IP1 knockdown alone suppressed clonogenic growth, and enhanced TQ’s inhibitory effect; conversely, PDZK1IP1 overexpression partially rescued growth in TQ-treated cells (Figure 5B). EdU incorporation assays gave consistent results: PDZK1IP1 silencing exacerbated TQ-induced reduction of EdU-positive cells, while its overexpression partially restored proliferation in the presence of TQ (Figure 5C).

**FIGURE 5:**
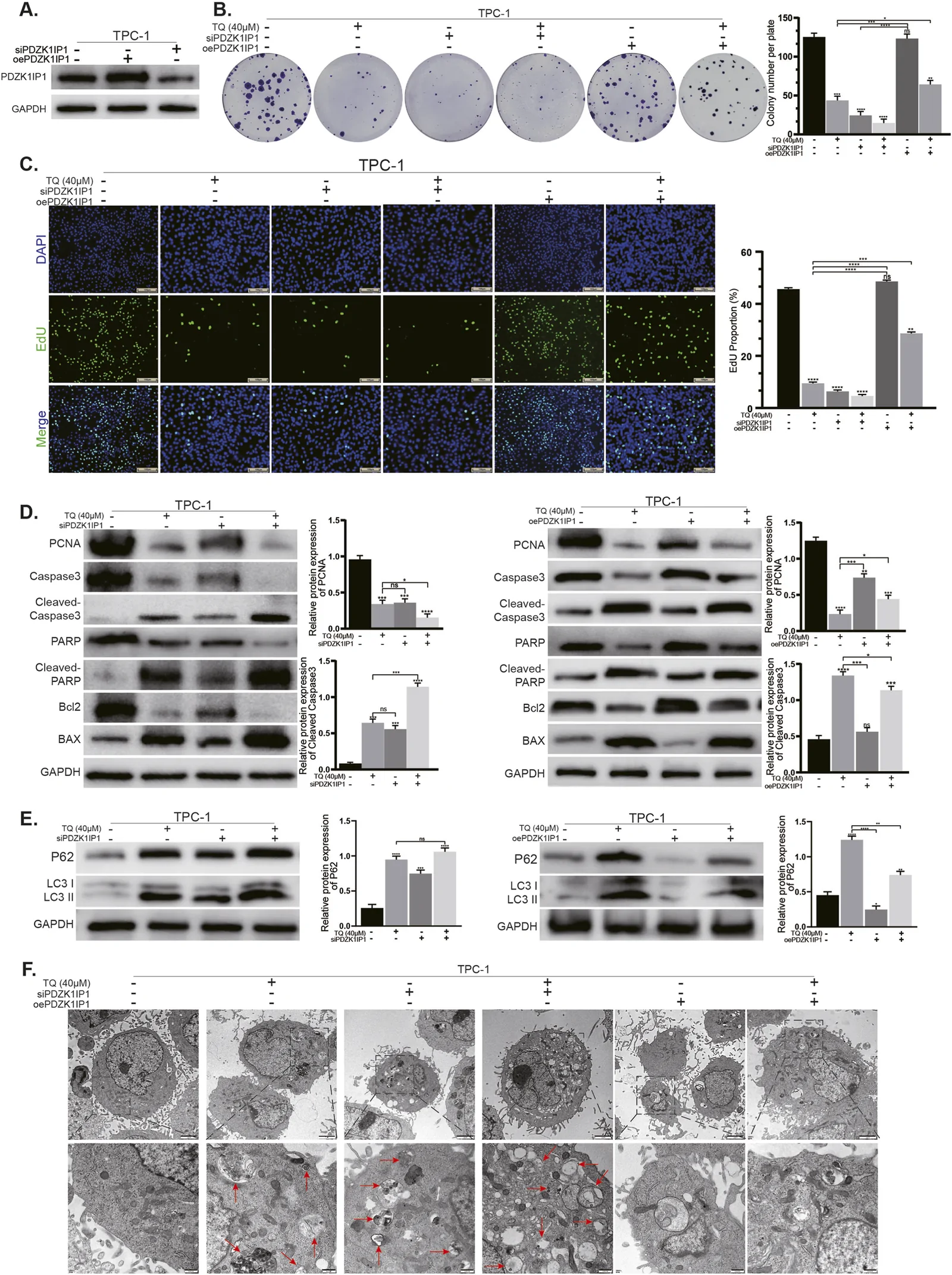
PDZK1IP1 mediates TQ’s effect on proliferation, apoptosis, and autophagy in TPC-1 cells. **(A)** Western blot verification of PDZK1IP1 knockdown and overexpression. **(B)** Colony formation assays as indicated. **(C)** EdU incorporation assays as indicated. **(D)** Western blot analysis of proliferation- and apoptosis-related proteins. **(E)** Western blot analysis of p62 and LC3. **(F)** TEM of ultrastructural changes after PDZK1IP1 silencing or overexpression with or without TQ treatment.

Western blot analysis confirmed these phenotypic changes at the molecular level. In TQ-treated cells, PDZK1IP1 knockdown further downregulated PCNA and Bcl-2, while upregulating cleaved caspase-3, cleaved PARP, and Bax; PDZK1IP1 overexpression largely attenuated these TQ-induced changes (Figure 5D). For autophagy markers, PDZK1IP1 silencing augmented TQ-induced accumulation of p62 and LC3-II, whereas its overexpression blunted these increases (Figure 5E). TEM imaging further validated these findings: TQ treatment or PDZK1IP1 knockdown alone induced robust accumulation of vacuolar and autophagosome-like structures, an effect markedly attenuated by PDZK1IP1 overexpression (Figure 5F). Collectively, these data demonstrate that PDZK1IP1 is a key mediator of TQ-induced proliferation inhibition, apoptosis induction, and autophagic flux impairment in PTC cells.

### 3.6 HMGCS1 acts downstream of PDZK1IP1 to mediate the effects of TQ in PTC cells

To identify downstream effectors of PDZK1IP1 in the TQ response, we performed Gene Ontology (GO) and Kyoto Encyclopedia of Genes and Genomes (KEGG) enrichment analysis on differentially expressed genes (DEGs) between control and high-dose TQ-treated TPC-1 cells. Enrichment analysis revealed significant overrepresentation of metabolism-related biological processes and pathways, including small molecule biosynthetic processes (GO), as well as butanoate metabolism and steroid biosynthesis (KEGG; Figure 6A). Based on these results and its expression pattern in our RNA-seq data, we selected 3-hydroxy-3-methylglutaryl-CoA synthase 1 (HMGCS1), a key metabolic enzyme, as a downstream candidate for further investigation.

**FIGURE 6:**
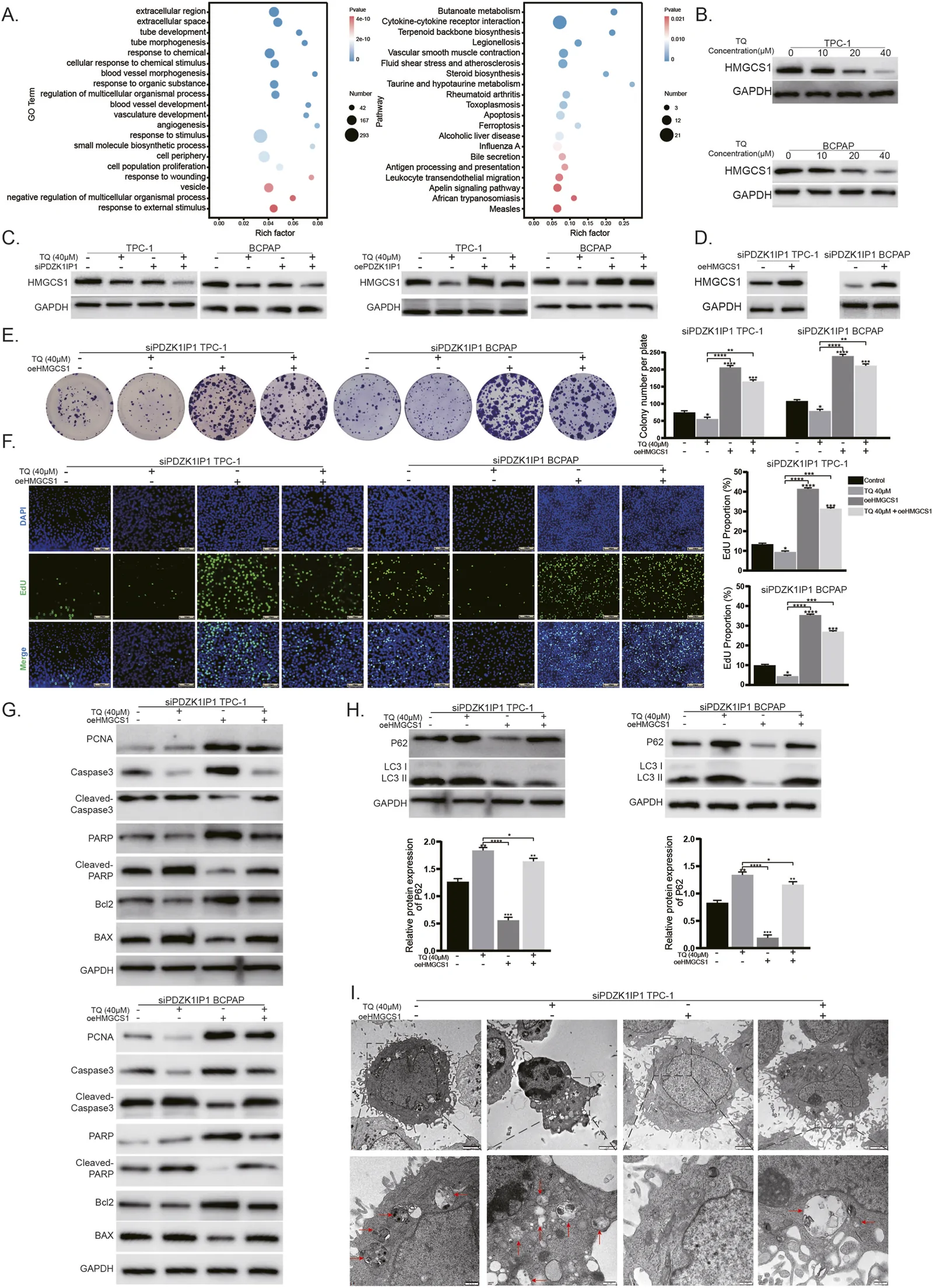
HMGCS1 functions downstream of PDZK1IP1 in TQ-treated PTC cells. **(A)** GO and KEGG enrichment analyses of differentially expressed genes (DEGs) between NC and high-dose TQ groups. **(B)** Western blot analysis of HMGCS1 expression in TPC-1 and BCPAP cells treated with increasing concentrations of TQ. **(C)** Western blot analysis of HMGCS1 after PDZK1IP1 knockdown or overexpression in TPC-1 and BCPAP cells. **(D)** Western blot verification of HMGCS1 overexpression in PDZK1IP1-silenced TPC-1 and BCPAP cells. **(E)** Colony formation assays in PDZK1IP1-silenced TPC-1 and BCPAP cells with or without TQ and HMGCS1 overexpression. **(F)** EdU incorporation assays under the same conditions. **(G)** Western blot analysis of proliferation- and apoptosis-related proteins in TPC-1 and BCPAP cells. **(H)** Western blot analysis of p62 and LC3 in TPC-1 and BCPAP cells. **(I)** TEM of ultrastructural changes in TPC-1 cells after HMGCS1 overexpression under the indicated conditions.

Western blot analysis revealed that HMGCS1 was dose-dependently downregulated by TQ in both TPC-1 and BCPAP cells (Figure 6B). In both cell lines, PDZK1IP1 knockdown reduced HMGCS1 expression, whereas PDZK1IP1 overexpression partially restored HMGCS1 levels in TQ-treated cells (Figure 6C). Efficient HMGCS1 overexpression in PDZK1IP1-silenced cells was confirmed in both cell lines by western blot (Figure 6D). These findings indicate that HMGCS1 is positively regulated by PDZK1IP1 and likely functions as a downstream effector of the PDZK1IP1 axis in TQ-treated PTC cells.

### 3.7 HMGCS1 overexpression partially rescues the phenotypic changes induced by TQ treatment and PDZK1IP1 silencing

To validate the functional role of HMGCS1 in TQ-induced and PDZK1IP1-mediated phenotypic changes, we performed parallel rescue experiments in TPC-1 and BCPAP cells. Under PDZK1IP1-silenced conditions, TQ further suppressed colony formation, whereas HMGCS1 overexpression partially restored clonogenic growth in the absence and presence of TQ in both cell lines (Figure 6E). EdU incorporation assays gave consistent results: HMGCS1 overexpression increased the proportion of EdU-positive cells under both conditions in TPC-1 and BCPAP cells (Figure 6F).

Western blot analysis further validated these rescue effects at the molecular level. In both cell lines under PDZK1IP1-silenced conditions, HMGCS1 overexpression increased PCNA and Bcl-2 and reduced cleaved caspase-3, cleaved PARP, and Bax in the absence and presence of TQ (Figure 6G). HMGCS1 overexpression also attenuated the accumulation of p62 and LC3-II in TPC-1 and BCPAP cells (Figure 6H). TEM imaging in TPC-1 cells showed that the accumulation of vacuolar and autophagosome-like structures induced under the same conditions was diminished following HMGCS1 overexpression (Figure 6I). Taken together, these parallel results demonstrate that HMGCS1 functions downstream of PDZK1IP1 to mediate TQ-induced regulation of proliferation, apoptosis, and autophagic processing in PTC cells.

### 3.8 TQ suppresses PTC tumor growth *in vivo* by regulating the PDZK1IP1/HMGCS1 axis and autophagic flux

Finally, we validated the *in vivo* antitumor activity of TQ using a subcutaneous PTC xenograft mouse model. Gross examination revealed that tumors from TQ-treated mice were markedly smaller than those from vehicle-treated controls, with a corresponding reduction in tumor weight at endpoint (Figure 7A). Two-way repeated-measures ANOVA showed a significant treatment effect on tumor volume (F(1, 10) = 27.77, P = 0.00036) and a significant treatment-by-time interaction (Greenhouse-Geisser-adjusted P < 0.0001). Sidak-adjusted comparisons showed lower tumor volumes in the TQ group on days 15, 18, and 21 (all P < 0.0001; Figure 7B). For body weight, neither the treatment effect (F(1, 10) = 1.40, P = 0.265) nor the treatment-by-time interaction (Greenhouse-Geisser-adjusted P = 0.0808) was significant, and no between-group difference was significant at any individual time point (all Sidak-adjusted P > 0.05; Figure 7C).

**FIGURE 7:**
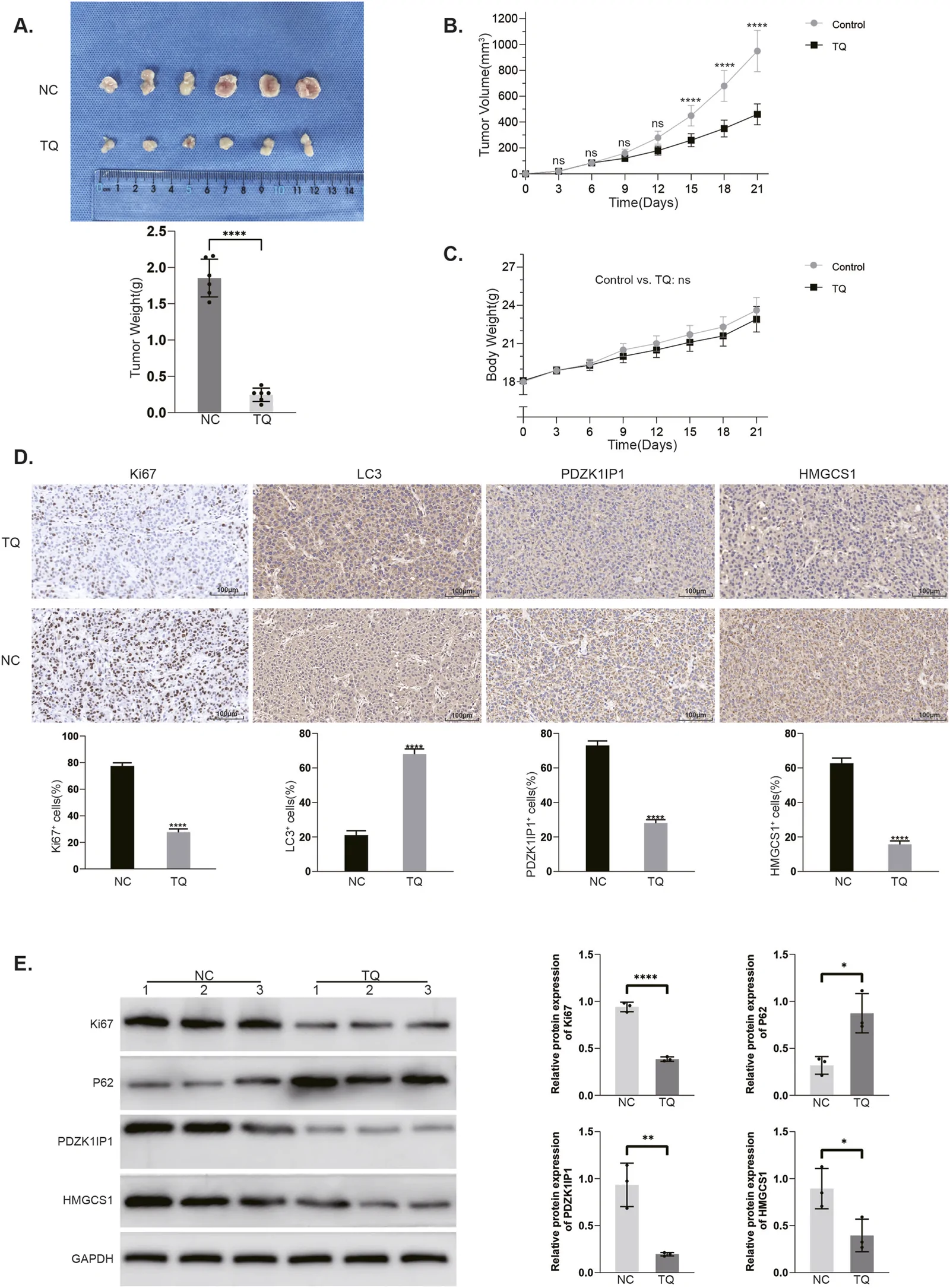
TQ suppresses tumor growth *in vivo* and reduces PDZK1IP1 and HMGCS1 expression. **(A)** Representative images of xenograft tumors and quantification of tumor weights. **(B)** Tumor growth curves during treatment. **(C)** Body weight curves of mice in the control and TQ-treated groups. **(D)** Immunohistochemical staining and quantification of Ki67, LC3, PDZK1IP1, and HMGCS1 in xenograft tissues. **(E)** Western blot analysis of Ki67, p62, PDZK1IP1, and HMGCS1 in xenograft tissues. For **(B)** and **(C)**, data are presented as mean ± SD (n = 6 mice per group). Longitudinal tumor-volume and body-weight data were analyzed using two-way repeated-measures ANOVA, with treatment as the between-subject factor and time as the within-subject factor. The Greenhouse-Geisser correction was applied to the repeated time factor, followed by Sidak’s multiple-comparisons test comparing the control and TQ groups at each time point. For endpoint two-group comparisons in **(A)**, **(D)**, and **(E)**, an unpaired Student’s t-test was used. *P < 0.05, **P < 0.01, and ****P < 0.0001; ns, not significant.

Immunohistochemical (IHC) staining of xenograft tissues recapitulated the *in vitro* findings: TQ treatment reduced the fraction of Ki67-positive proliferating cells, downregulated PDZK1IP1 and HMGCS1, and increased LC3 staining compared to control tumors (Figure 7D). Western blot analysis of xenograft lysates further confirmed these results: TQ downregulated Ki67, PDZK1IP1, and HMGCS1, while upregulating the autophagic flux marker p62 (Figure 7E). Collectively, these data demonstrate that TQ suppresses PTC tumor growth *in vivo* through downregulation of the PDZK1IP1/HMGCS1 axis and impairment of autophagic flux.

## 4 Discussion

Papillary thyroid carcinoma (PTC) remains challenging when disease recurs, metastasizes, or becomes treatment refractory. In this study, thymoquinone (TQ) inhibited proliferation and promoted apoptosis in TPC-1 and BCPAP cells, while also suppressing PTC xenograft growth. These effects were accompanied by reduced PCNA and Bcl-2 and increased cleaved caspase-3, cleaved PARP, and Bax. The results extend previous evidence of TQ activity in solid malignancies (Ozturk et al., 2018; Zhou et al., 2021). They also identify impaired autophagic processing and downregulation of the PDZK1IP1/HMGCS1 axis as linked components of the response in PTC.

Autophagy is dynamic, so LC3-II accumulation alone cannot distinguish increased autophagosome formation from impaired degradation (Dong et al., 2023; Muralidharan et al., 2021). We therefore combined ultrastructural imaging, tandem mRFP-GFP-LC3B analysis, biochemical markers, and pharmacological modulation. TQ increased autophagosome-like vesicles, yellow and red-only LC3 puncta, and p62 and LC3-II accumulation. CQ further enhanced the antiproliferative and proapoptotic effects of TQ, whereas RAP partially attenuated them (Deng et al., 2019; He et al., 2023). These findings support disturbed late-stage autophagic processing rather than isolated activation of autophagy initiation. This interpretation is consistent with the stress-adaptive role of intact autophagic flux in cancer cells (Chryplewicz et al., 2022; Ferro et al., 2020). However, the observed increase in red-only puncta indicates that maturation was not completely abolished. The current data cannot distinguish defective autophagosome-lysosome fusion from altered lysosomal acidification or proteolytic activity (Assi et al., 2026; Chen P. et al., 2024).

Integrated analysis of public PTC datasets and RNA sequencing of TQ-treated TPC-1 cells identified *PDZK1IP1* and *GBP2* as candidates with opposing disease- and treatment-associated expression patterns. PDZK1IP1 was prioritized because TQ consistently reduced its protein abundance in both PTC cell lines. PDZK1IP1 knockdown enhanced TQ-induced growth inhibition, apoptosis, and autophagy-marker accumulation, whereas overexpression partially attenuated these effects. The incomplete rescue indicates that PDZK1IP1 contributes to, but does not fully account for, the activity of TQ (Deng et al., 2020; Chen X. et al., 2024). PDZK1IP1, also known as MAP17, has context-dependent roles in tumor progression, redox regulation, and treatment response (Khan et al., 2024; Hsiao et al., 2023). Our findings extend this literature by linking PDZK1IP1 to TQ responsiveness and altered autophagic processing in PTC, although the mechanism connecting PDZK1IP1 to the autophagy machinery remains unresolved.

Pathway enrichment led us to investigate HMGCS1, an upstream enzyme in the mevalonate pathway. HMGCS1 supports tumor-cell biosynthesis and proliferation (Dai et al., 2025; Li et al., 2024; Yang et al., 2023). More broadly, autophagy-derived metabolites can influence tumor progression, illustrating functional crosstalk between autophagy and cancer metabolism (Han et al., 2022). TQ reduced HMGCS1 expression in both PTC cell lines. PDZK1IP1 knockdown produced a similar effect, whereas PDZK1IP1 overexpression partially restored HMGCS1 after TQ treatment. HMGCS1 overexpression also partially rescued proliferation and attenuated apoptosis and autophagy-marker changes in PDZK1IP1-silenced TPC-1 and BCPAP cells. These results place HMGCS1 downstream of PDZK1IP1 but also indicate that additional mechanisms contribute to the TQ response (Chen L. et al., 2024; Xia et al., 2025). Other mevalonate-pathway components may participate. HMGCR is the canonical rate-limiting enzyme, while mevalonate kinase supports subsequent sterol and non-sterol isoprenoid synthesis (Mullen et al., 2016). TQ can reduce HMGCR expression in HepG2 cells, and HMGCR inhibition disrupts malignant phenotypes in anaplastic thyroid cancer cells (Al-Naqeep et al., 2009; Zhong et al., 2005). The roles of HMGCR, mevalonate kinase, and downstream metabolites in TQ-treated PTC require direct testing.

The xenograft findings support the relevance of the cellular results *in vivo*. TQ reduced tumor growth without a significant between-group difference in body weight at the tested dose. Xenograft tissues also showed reduced Ki67, PDZK1IP1, and HMGCS1, together with increased LC3 and p62. These changes were consistent with the antiproliferative, pathway-related, and autophagy-associated effects observed *in vitro* (El-Far et al., 2021; Rachamalla et al., 2021). Nevertheless, this model does not establish clinical efficacy or exclude toxicities that were not captured by body-weight monitoring.

Several limitations define the scope of these conclusions. Additional PTC models, including patient-derived organoids, are needed to establish generalizability. Lysosomal pH, proteolytic activity, and fusion dynamics should be measured to identify the precise late-stage autophagic defect. The regulatory mechanism linking PDZK1IP1 to HMGCS1 also remains unknown. Moreover, the genetic and rescue data demonstrate functional mediation but not direct binding of TQ to PDZK1IP1, HMGCS1, or autophagy-related proteins. Although TQ can bind other proteins through noncovalent or covalent mechanisms (Yin et al., 2013; Acharya et al., 2014; Gil et al., 2017), direct targets in PTC require validation. Molecular docking should therefore be combined with surface plasmon resonance and cellular thermal shift assays in future studies. Finally, PDZK1IP1 and HMGCS1 require validation in human PTC specimens. Within these boundaries, our results support a model in which TQ suppresses PTC growth by disturbing late-stage autophagic processing and downregulating the PDZK1IP1/HMGCS1 axis.

## Data Availability

The datasets presented in this study can be found in online repositories. The names of the repository/repositories and accession number(s) can be found below: https://figshare.com/, 10.6084/m9.figshare.33121082 https://www.ncbi.nlm.nih.gov/geo/, GSE138198, GSE27155, GSE33630, GSE9115.
